# *“In medio stat virtus”*: Insights into hybrid E/M phenotype attitudes

**DOI:** 10.3389/fcell.2022.1038841

**Published:** 2022-11-18

**Authors:** Angelo Canciello, Adrián Cerveró-Varona, Alessia Peserico, Annunziata Mauro, Valentina Russo, Andrea Morrione, Antonio Giordano, Barbara Barboni

**Affiliations:** ^1^ Faculty of Bioscience and Technology for Food Agriculture and Environment, University of Teramo, Teramo, Italy; ^2^ Department of Biology, College of Science and Technology, Temple University, Philadelphia, PA, United States; ^3^ Sbarro Health Research Organization (SHRO), Philadelphia, PA, United States; ^4^ Department of Medical Biotechnologies, University of Siena, Siena, Italy

**Keywords:** hybrid E/M phenotype, partial EMT, stem cell, cancer, immune evasion, immune suppression, collective migration, metabolic reprograming

## Abstract

Epithelial-mesenchymal plasticity (EMP) refers to the ability of cells to dynamically interconvert between epithelial (E) and mesenchymal (M) phenotypes, thus generating an array of hybrid E/M intermediates with mixed E and M features. Recent findings have demonstrated how these hybrid E/M rather than fully M cells play key roles in most of physiological and pathological processes involving EMT. To this regard, the onset of hybrid E/M state coincides with the highest stemness gene expression and is involved in differentiation of either normal and cancer stem cells. Moreover, hybrid E/M cells are responsible for wound healing and create a favorable immunosuppressive environment for tissue regeneration. Nevertheless, hybrid state is responsible of metastatic process and of the increasing of survival, apoptosis and therapy resistance in cancer cells. The present review aims to describe the main features and the emerging concepts regulating EMP and the formation of E/M hybrid intermediates by describing differences and similarities between cancer and normal hybrid stem cells. In particular, the comprehension of hybrid E/M cells biology will surely advance our understanding of their features and how they could be exploited to improve tissue regeneration and repair.

## Complexity of EMT/MET programs regulation

Epithelial-mesenchymal transition (EMT) is complex process thereby epithelial cells lose their characteristics along with native phenotype and the apical-basal polarity to acquire mesenchymal features including front-back polarity and migratory capacity. EMT is a completely reversible process; therefore, mesenchymal cells can revert and back epithelial again through mesenchymal-epithelial transition (MET) (Bakir et al., 2020). This trans-differentiation is involved in a number of biological processes, including, among the others, embryogenesis, stemness (and re-activation), cell differentiation, tissue regeneration, wound healing, fibrosis, cancer stem cell reprogramming and generation, metastasis and migration, metabolic reprogramming, immune evasion and chemotherapy resistance (T. Chen et al., 2017; M. Singh et al., 2018; Georgakopoulos-Soares et al., 2020; S. Brabletz et al., 2021). Indeed, it is easily understandable why such a pleiotropic process has become source of intense study by scientists from very different fields.

At the lead of the transdifferentiating process there is a tangled womb of finely regulated cellular and molecular events which orchestrate EMT. In particular, EMT is governed by a multitude of factors which including extracellular matrix (ECM) components, hypoxia, exosomes, non-coding RNAs, cytokines and growth factors such as hepatocyte growth factor (HGF), fibroblast growth factor (FGF) and transforming growth factor-β (TGFβ), Wnt, Notch, Hedgehog and many others (Chaffer et al., 2016). Ultimately, these signals lead to the activation of various master regulators of EMT program, specifically EMT-inducing transcription factors (EMT-TFs) like members of the SNAIL family (SNAIL, and SLUG), TWIST family of basic helix–loop–helix factors (TWIST1 and TWIST2), Zinc-finger E-box-binding homeobox (ZEB) factors (ZEB1 and ZEB2), Paired-related homeobox 1 (PRRX1), Forkhead box C2 (FOXC2) and YAP/TAZ (Stemmler et al., 2019). On the other hand, different TFs such as Ovo-like transcriptional repressor (OVOL1 and OVOL2), Grainyhead-like 2 (GRHL2), E74-like ETS transcription factor 5 (ELF5), KLF4 and p53 counterbalance EMT-TFs, thereby the maintaining of cells in an epithelial state or, in some cases, inducing MET (Sinha et al., 2020; Deshmukh et al., 2021). The EMT-TFs trigger the key step of EMT program: the disruption of adherent and tight junctions of epithelial (E) cells and the consequent reorganization of actin fibers with the aggravation of mesenchymal (M) markers expression (conventionally represented by the loss of E-cadherin and the gain of N-cadherin expression) (Gonzalez and Damian, 2014). Additional levels of regulation are provided by certain miRNAs (e.g. miR34, miR200) as well as post-transcriptional regulation of splicing (e.g. ESRP1) and post-translational control of protein stability (e.g. ubiquitination and degradation of SNAIL) (Aiello and Kang 2019; Lambert and Weinberg 2021).

## EMP governs the fates of hybrid E/M states

**FIGURE 1:**
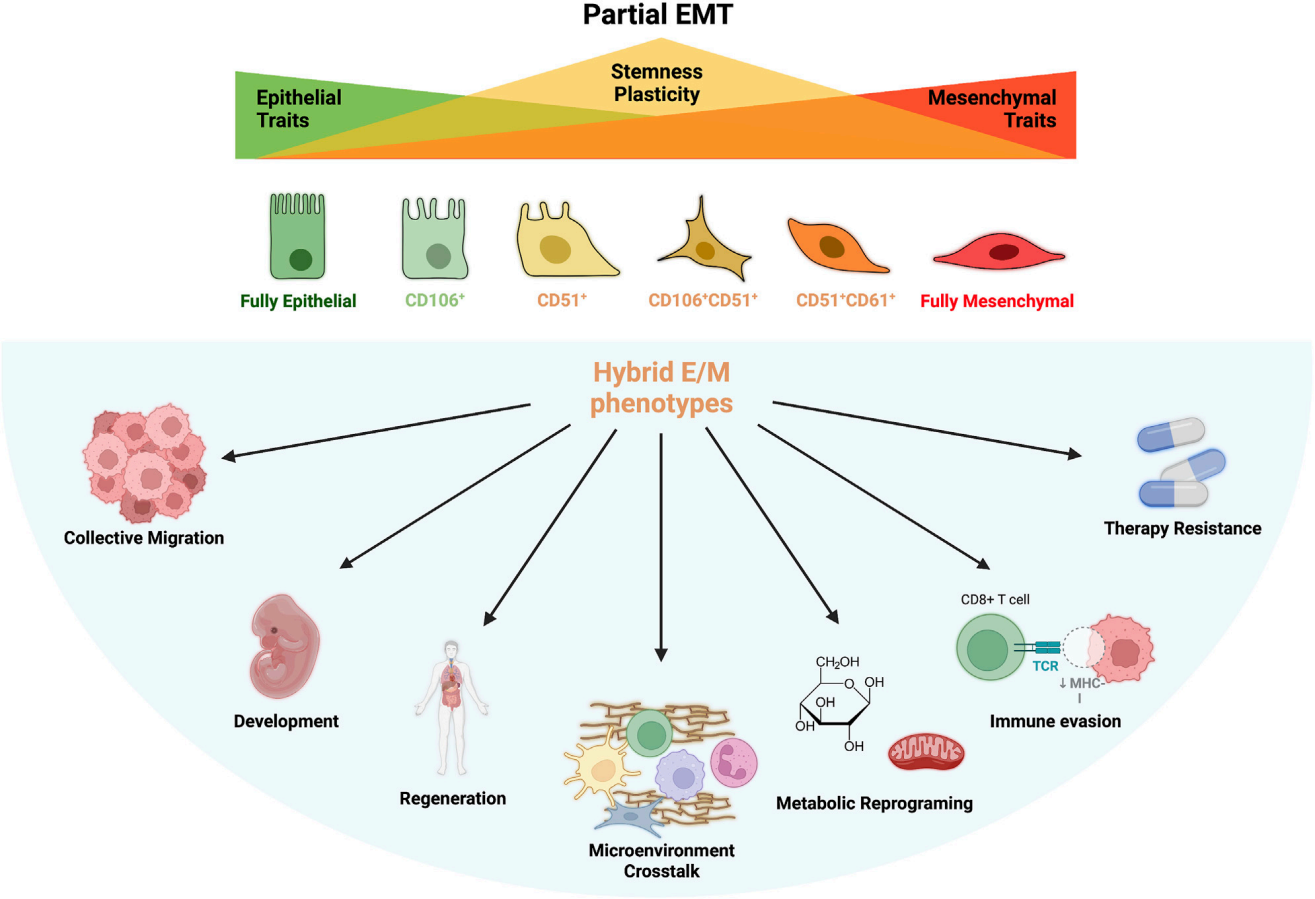
Partial EMT and Hybrid E/M Phenotypes. During partial EMT are generated several intermediated phenotypes which bear mixed epithelial (E) and mesenchymal (M) features. These states are called hybrid E/M phenotype and display particular surface markers such as CD106, CD51 and CD61. The loss of E phenotype coincides with an increase of stemness and plasticity that reaches its peak during the formation of hybrid states and decrease again when EMT is complete. Plasticity and stemness are at the base of hybrid cells peculiar features: collective migration, development and differentiation, regeneration, microenvironmental organization, metabolic plasticity, immune evasion, immune suppression and therapy resistance.

Recent findings derived by mathematical modelling of this multilayered regulatory network successively supported by numerous biological observations, provided new insight: EMT or MET are not “all-or-none” processes but rather they generate cells residing in a number of intermediate hybrid E/M states lying between the fully E and fully M poles (Zhang et al., 2014; Pastushenko et al., 2018; Gómez Tejeda et al., 2019; Jia et al., 2019; Xing and Tian 2019; Pasani et al., 2021) (Figure 1). Therefore, it becomes evident as the “EMT/MET dichotomy” seldom represents a practically applicable mechanism in complex biological contexts (Jolly et al., 2015a; Kröger et al., 2019; Bornes et al., 2021). Indeed, it is widely accepted that partial EMT or MET normally occur during early embryonic development, as well as during wound healing in adults (Plygawko et al., 2020; Marconi et al., 2021). Moreover, E/M intermediates generated from incomplete EMT/MET are also known to participate in cancer initiation and metastatic process (Jolly et al., 2019; Pastushenko and Blanpain 2019; Sacchetti et al., 2021). However, a salient characteristic of *in vivo* EMT, either during normal development and in a pathological context, is that the transition from E to M state is very often incomplete, resulting in cells that reside in intermediate states that retain both E/M characteristics (Lambert and Weinberg 2021). Moreover, these (discrete or fluid) intermediate hybrid states are dynamically interconnected and bear huge plastic ability to move readily among these E/M intermediates or, alternatively, toward a fully E or fully M state, though the extent to which such intermediates represent metastable states in specific biological contexts is unclear (Jolly et al., 2016; Deshmukh et al., 2021). Therefore, the term epithelial-mesenchymal plasticity (EMP) adopted to describe this phenomenon (Yang et al., 2020).

Increasing evidences effectively demonstrated the involvement of EMP in development, wound healing, cancer and, regeneration and whose processes are mostly mediated by cells with hybrid E/M phenotype expressing peculiar functional characteristic (B. Huang et al., 2015) (Figure 1). Nonetheless, hybrid E/M phenotype is associated with E cell reprograming accompanied by the upregulation of stemness genes (e.g. Oct4, Sox2, Nanog) which is the base for the formation of cancer stem cells (CSC) in tumor bulk (Jolly et al., 2015b). Moreover, loss of adherent junction and simultaneous expression of integrins for ECM interactions increases cell survival of hybrid E/M cells and activate anoikis program through which E cells evade death induced by the detachment form basal membrane (Sinha et al., 2020). A favorable metabolic reprogramming—an emerging hallmark of cancer cells—was also observed in hybrid E/M cells which use both oxidative phosphorylation (OXPHOS) and glycolysis for ATP production, irrespective of the presence of oxygen (also known as Warburg effect or aerobic glycolysis), thus improving self-renewal capacity in hypoxic niches (Jia et al., 2021) (Figure 1).

The present review aims to describe the main features and the molecular events regulating EMP and the formation of E/M hybrid intermediates. In particular, differences and similarities between the cancer and normal hybrid stem cells will be disserted. Furthermore, particular emphasis will be addressed to summarize the evidences collected to date of the mechanisms accomplished by E/M hybrid intermediates in order to develop a more performant phenotype with enhanced stemness/migration properties, activation of O_2_-independent metabolic pathways and increased immune privilege, thus escaping from neighbor immune cells surveillance.

### Markers of hybrid E/M states

Cells does not toggle between alternative E and M states. Rather, EMT generates a diverse array of hybrid cells bearing various degrees of both E and M features. It still remains unclear whether discrete phenotypic states are arrayed along E/M phenotypic spectrum or, alternatively, there is a continuum of such states lacking of distinct and definable boundaries. Several research groups tried to untie the puzzle by using different approaches. To this regard, Pastushenko and others (2018) screened E cells (Epcam^+^/K14^+^/VIM^−^) and cells undergoing EMT program (Epcam^−^/K14^low/-^/VIM^+^)—isolated from skin squamous cell carcinoma (SCC)—for a large panel of surface markers, seeking for those populations that displayed a heterogeneous expression. As a consequence, six distinct populations of hybrid E/M phenotypes have been identified. Starting from the same dataset of SCC, another research group was able to identified 4 cell types (E/M states) with the aid of bioinformatic analyses based on gene expression clustering (Bocci et al., 2021). Intriguingly, in an previous study RNA–in situ hybridization (ISH) was used to examine the expression of E and M transcripts of either primary tumor cells and circulating tumor cells (CTCs) isolated from breast cancer patients, leading to the classification of five different phenotypes (E, M and three intermediated E/M states) (Yu et al., 2013). Considering the complexity of the process and the fact that the identification of discrete E/M phenotypes mostly rely on the methods and the markers (and perhaps the origin of the specimen) used for the analysis, it is still difficult to claim the precise number (if any) of intermediated states occurring during EMT. Moreover, most of the published articles on this subject identified as hybrid E/M cells those which display simultaneously mixed E and M markers. However, much more effort should be made to the identification of univocal hybrid E/M markers able to selectively and unambiguously isolate those populations, even though the great heterogeneity reported made the goal extremely challenging. Indeed, a multistep-multilayered approach that sequentially evaluate surface markers and transcription/proteomic signature together with functional assays could represent a possible starting point.

To this regard, hybrid E/M intermediates are characterized by different extent of E and M markers co-expression. In particular, hybrid E/M cells were found to share the expression of typical E markers, such as E-cadherin, Epcam, Cytokeratins (Krt5, Krt8, Krt14) along with M proteins including Vimentin, N-Cadherin and α-SMA (Sinha et al., 2020). The identification of hybrid E/M through the expression of mixed E/M markers has been associated in cancer cells with a poor prognosis and a higher risk metastatic process rather than fully M cells (J. Zhang et al., 2014; Pastushenko et al., 2018; Kröger et al., 2019; Carstens et al., 2021; Aggarwal et al., 2021). Furthermore, other surface markers such as placental-cadherin (P-cadherin), Slug and, integrin-β 4 (ITGB4 or CD104) were found particularly expressed in hybrid E/M cells. Intriguingly, P-cad and Slug are indicated to play pivotal roles in collective cell migration, a specific features of hybrid E/M cells (Liao and Yang 2020; Noronha et al., 2021). Noteworthy, three specific proteins including regulator of G-protein signaling 16 (RGS16), plasminogen activator inhibitor-2 (Pai 2, also known as SerpinB2) and an integrin α3 (ITGA3) were reported to be particularly upregulated in hybrid E/M cells while they were not expressed by neither E or M cells, individually (Saitoh 2018).

Cancer cells within individual carcinomas often exhibit phenotypic heterogeneity in terms of E and M markers expression. To this regard, CD24 and CD44 surface markers have been widely used to distinguish between E (CD24) and M (CD44) cancer cells (Nieto et al., 2016). Interestingly, CD24/CD44 co-expression has been related in tumor cells to an increased stemness and specifically by identifying the subset of tumor-initiating cells also known as cancer stem cells (see below). To this regards, using flow cytometry Grosse-Wilde *et al.* isolated a subpopulation of CD24^+^CD44^+^ breast cancer cells also characterized by the mixed expression of E/M genes (such as E-cadherin, Epcam, VIM and ZEB), thus demonstrating that CD24^+^CD44^+^ co-expression is an hallmark of the hybrid E/M phenotype (Grosse-Wilde et al., 2015). However, it has recently been reported that the expression of CD104 (known as integrin β4 or ITGB4) is another characteristic surface antigen of hybrid E/M states (Bierie et al., 2017; Kröger et al., 2019). In particular, increasing evidences indicate that the differential expression between CD104/CD44 allows to divide the phenotype EMP stepwise process: E (CD104^+^CD44^lo^), hybrid E/M (CD104^+^CD44^hi^), and M (CD104^−^CD44^hi^) (Bierie et al., 2017).

An increasing amount of experimental evidences have proved the existence also of specific marker profiles to follow the hybrid E/M intermediates during the EMP progression (Pastushenko et al., 2018) (Figure 1). More in detail, CD51 (ITGAV), CD61 (ITGB3) and CD106 (VCAM-1) expressions can be used as indicative of the early and late hybrid E/M states. To this regard, early hybrid E/M phenotype has been tentatively associated with the 1) triple negative (or TN: CD51^−^CD61^−^CD106^−^), 2) CD106^+^ or 3) CD51^+^ subpopulations which exhibit co-expression K14 and VIM. Conversely, late hybrid more M-like phenotypes can be identify with the 4) CD106^+^CD51^+^, 5) CD51^+^CD61^+^ and the 6) triple positive (or TP: CD51^+^CD61^+^CD106^+^) subpopulations which are VIM^+^ and K14^-^ (Pastushenko et al., 2018) (Figure 1). Intriguingly, the hybrid TN and CD106^+^ subpopulations significantly generate more metastases compared to other subpopulations, further suggesting as the hybrid more than the fully M states are responsible for tumor malignancy and spreading (Pastushenko and Blanpain 2019).

### Stability and plasticity of hybrid E/M phenotype

The regulation of hybrid E/M states is subjected to a fine balance that allows not only the progression back and forth towards a fully E or M state but also the stabilization of hybrid E/M intermediates. As described below, a plethora of molecular factors govern EMP and all together form the so-called gene regulatory networks (GRNs) consist of miRNAs (miRs), TFs, alternative spicing factors, epigenetic modifiers, growth factors, long non-coding RNAs (lncRNA) and other proteins. Besides, another group of proteins defined as phenotypic stability factors (PSFs) are implied in the determination and maintaining of stable hybrid E/M states. In the following section the complexity of GRNs and PSFs connection and their functions will be discussed by depicting the molecular mechanisms that regulate stemness, migratory and immune plasticity of hybrid E/M cells.

### Gene regulatory networks regulate EMP

**FIGURE 2:**
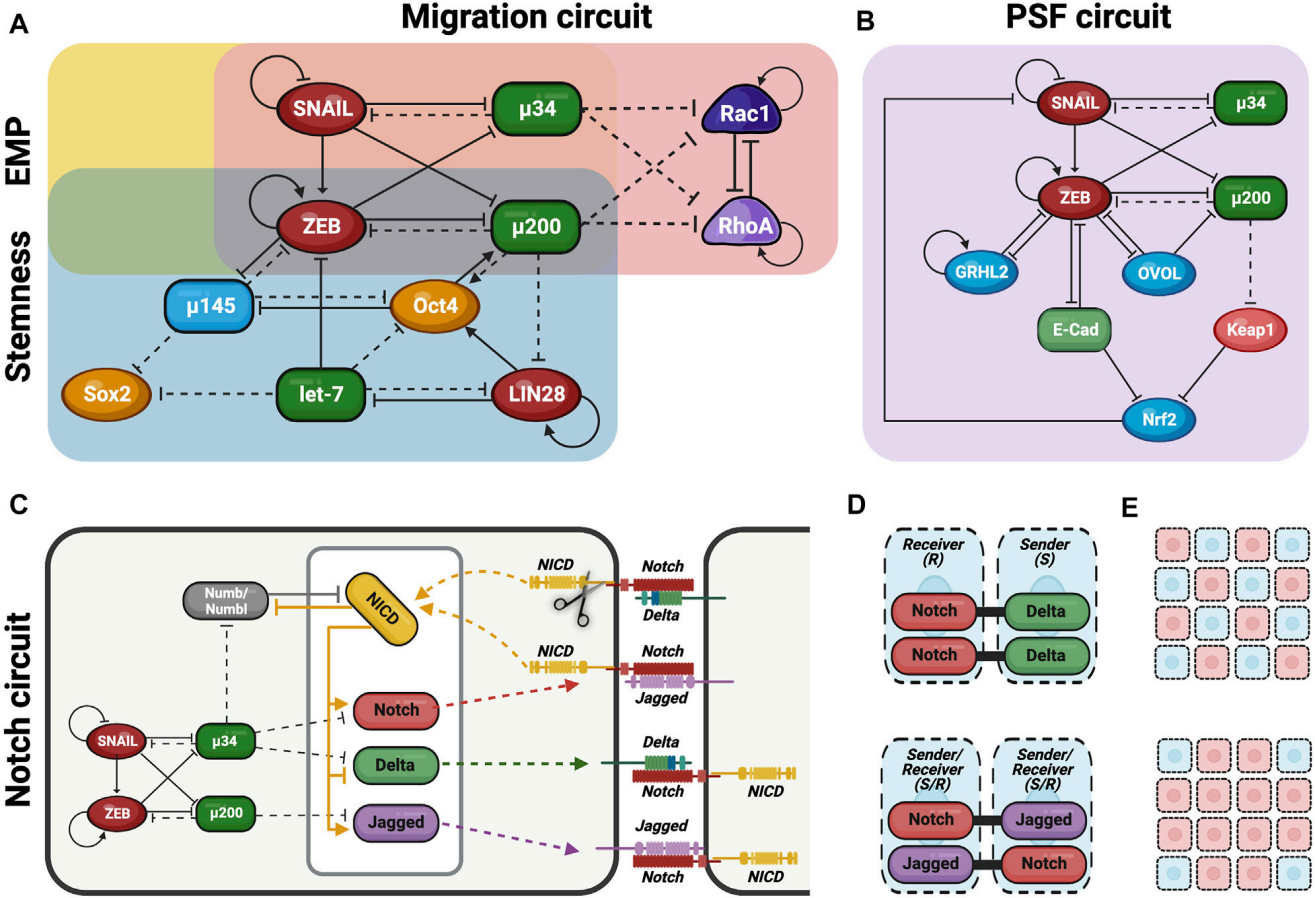
Gene regulatory networks (GRN) govern the fate of hybrid E/M cells. Schematic overview of the different GNR involved in the modulation of hybrid E/M phenotype decisions. **(A)** EMP circuit (yellow) is formed by the interaction of two mutually inhibitory feedback loops (miR-200/ZEB and miR-34/SNAIL) and regulates EMT/MET decisions. Stemness circuit (blue) integrate EMP circuit with another mutually inhibitory feedback loop formed by LIN28/let-7 and regulates the expression of stemness genes. Migration circuit (red) involves Rac1/RhoA interactions and is also integrated with EMP circuit thus regulating the switch between collective and individual migration. **(B)** Phenotypic Stability Factors (PSF) such as OVOL, GRHL2 and Nrf2 maintain the hybrid E/M phenotype, thus avoiding a complete EMT. PSF circuit (purple) is completely integrated in the EMP circuit and their interactions regulate the cell fate. **(C)** Coupling EMP circuit with Notch circuit (light green) which regulates cell-cell communication and cell asymmetry. **(D)** Notch-Delta signaling induce different fate in neighboring cells: one becomes Sender (high Delta/low Notch) and the other becomes Receiver (high Notch/low Delta). This behavior causes lateral inhibition and promotes the formation of the so-called “salt and pepper” pattern. Notch-Jagged signaling induces the same fate in neighboring cells which become Sender/Receiver (high Notch/high Jagged). This behavior causes lateral induction and promotes the formation of the similar pattern among neighboring cells. **(E)** Therefore, during lateral inhibition (up) cells in partial EMT might not be spatially close, thus promoting single cell asymmetry. Conversely, during lateral induction (down) cells in partial EMT can mutually stabilize the hybrid E/M phenotype, thus generating cluster symmetry (Figures 2C–E have been adjusted from Bocci et al., 2017).

Mathematical modelling has first predicted that EMT/MET may not be symmetric processes and cells may get interconverted in multiple intermediate phenotypes lying between the fully E and fully M poles. Such predictions have borne out by numerous biological observations and recent advancements have starting to explore the logic of GRNs controlling the different EMT states (Figure 2).

Several groups have proposed that the interactions among two microRNA families, miR-200 and miR-34, and two EMT-TFs, ZEB and SNAIL, form the EMT regulatory network core (Xing and Tian 2019; Hari et al., 2020; Pasani et al., 2021) (Figure 2A). In particular, these interactions form mutually inhibitory loops among them determining cell phenotype depending on which one prevail on the others. However, the mutually inhibitory loops may also promote a hybrid E/M phenotype in the absence of any strong inhibition/expression of the loop members (Pasani et al., 2021).

At this regard, E/M plasticity is in part controlled by a mutually inhibitory feedback loop between ZEB and miR-200. In detail, miR-200 is particularly expressed in E cells and suppress the transcription of ZEB, thus preventing EMT or, alternatively, inducing MET (Figure 2A).

On the contrary, ZEB—which can also self-activate through ESRP1 and/or CD44/HA signaling pathways—is expressed in M cells and inhibits miR-200, thus promoting EMT (Lu et al., 2013; Stemmler et al., 2019; Drápela et al., 2020) (Figure 2A). According to this network and sustained by experimental evidences, the combination of miR-200/ZEB pattern of expressions give rise to three possible phenotypes: E (miR-200^high^ ZEB^low^), hybrid E/M (miR-200^medium^ ZEB^medium^) and M (miR-200^low^ ZEB^high^).

Linked to miR-200/ZEB regulatory network there is another mutually inhibitory feedback loop formed by miR-34/SNAIL which is involved in the determination of cell fate phenotype (Pasani et al., 2021) (Figure 2A). In particular, SNAIL is a well-known EMT-TF which promote the transition toward M phenotype; conversely, miR-34 family has SNAIL as one of its molecular targets, thus preventing EMT and preserving E phenotype (L. Zhang, Liao, and Tang 2019). Interestingly, SNAIL which self-inhibits transcriptionally, can influence the first network by activating ZEB expression and suppressing miR-200 transcription (Lu et al., 2013) (Figure 2A). Therefore, miR-200 and miR-34 function as epithelial gatekeepers and when expressed at elevated levels determine the E phenotype (miR-200^high^/ZEB^low^, miR-34^high^/SNAIL^low^). On the other hand, ZEB and SNAIL promote EMT and induce the trans-differentiation toward M phenotype (miR-200^low^/ZEB^high^, miR-34^low^/SNAIL^high^). Finally, a hybrid state can be achieved when miR-34/SNAIL network switches from miR-34^high^/SNAIL^low^ to miR-34^low^/SNAIL^high^, but the miR-200/ZEB circuit is maintained at miR-200^high^/ZEB^low^ (Pasani et al., 2021). In this case, the hybrid E/M state is permitted by the co-expression of strong E guardian (miR-200) along with SNAIL which confer the M drive. According to this model, miR-200/ZEB circuit function as a three-way decision-making switch whereas the miR-34/SNAIL circuit primarily functions as a noise-buffering integrator.

Although GRN provides a general key of interpretation for hybrid E/M states generation, specific gene interactions regulating the same process could sensibly vary in different contexts. Recently, data collected from scRNA-seq of tumor samples together with computational modelling have provided subtle features for the interpretation of the intertwined relations regulating GRN during EMT. In particular, according to Font-Clos and others the topology of the GRN represent an important determinant for cell phenotype (Font-Clos et al., 2018). Basically, cells can be found in an extremely large variety of E, M and intermediate states, each one forming a defined cluster. It has been determined that only the cells that are topologically at the “edges” of each individual cluster/state is particularly prone to external perturbations (Bocci et al., 2021). In this scenario, late hybrid E/M phenotypes are more susceptible to fluctuations and tend to complete EMT much more likely respect to early hybrid E/M states (that reside in the “core” of the cluster). As consequence, M cells that apparently completed EMT are more prone to moves cells into late hybrid E/M state, thus undergoing MET program (Font-Clos et al., 2018). However, such perturbations needs to simultaneously modulate multiple factors of the GRN to produce a consistent effect on cell phenotype, thus fostering the formation of intermediate E/M states. (Steinway et al., 2015).

Noteworthy, time series scRNA-seq analyses from several cancer cells have also demonstrated that different external signals—such as TGFβ, EGF, and TNF—are able to induce EMT by triggering divergent intracellular pathways, with peculiar timing and trajectories (Cook and Vanderhyden 2020; Deshmukh et al., 2021). Intriguingly, the removal of such signals from the system and the consequent triggering of MET is achieved by taking backward phenotypic trajectory that do not overlap with the one undertaken through forward transition (Ramirez et al., 2020). Therefore, it seems that cells decide to go along two different paths, passing through intermediated phenotypes that differ depending on the direction of the transition. Whether such behavior could be partly explained by the different temporal activation of the same intracellular pathways, it is also influenced by cell-cell communication. In fact, cells in hybrid E/M states that receive TGFβ stimuli from the environment, will send in turn TGFβ inputs to other cells, thus inducing EMT also in their neighbors (Sha et al., 2021). This happens because TGFβ triggers a synchronous response in the sending and receiver cells. Conversely, either EGF and TNF induction of EMT is not direct but rely on divergent signaling pathways that also regulate other mechanisms (Kang et al., 2013; Hayden and Ghosh 2014; Bocci et al., 2019a). As a result, cell-cell communication is impaired and EMT induction is asynchronous, thus resulting in hybrid E/M cells resisting to complete EMT (Sha et al., 2021). In accord with this finding, another group demonstrate that an increased cell-cell communication emerged as a general features of hybrid E/M states, independently of the specific pathway (Bocci et al., 2021).

### Linking E/M plasticity, stemness, and cancer

On the basis of evidences collected to date, the old view according to whom full EMT was associated with increased stemness has to be updated. Indeed, it is now largely demonstrated that stem-like properties are enhanced by the acquisition of hybrid E/M states during EMT/MET (Pasani et al., 2021). To this regard, cancer cells undergoing partial EMT (pEMT) acquire proliferation, survival and, invasiveness through cluster migration, all features mainly associated with an increased stemness which eventually lead to the formation of cancer stem cells (CSCs). In addition, a complete EMT is considered dispensable for acquiring stemness, representing instead an obstacle for its achievement (Grosse-Wilde et al., 2015; Pastushenko et al., 2018; Kröger et al., 2019; Carstens et al., 2021; Sacchetti et al., 2021). For instance, EMT has been long thought to be related to metastatic process favoring the migration of cancer cells toward a second site of colonization. However, this view needs to be updated with the evidence that the acquisition of a fully M state is instead associated with a reduced colonizing ability specifically due to the loss of stemness (Ocaña, 2012; Tsai et al., 2012). Indeed, the generation of metastable heterogeneous cancer phenotypes is likely at the base of metastasis and therapy resistance observed in different tumor (Brown et al., 2022). To this regard, it has been demonstrated that tumor cells infrequently undergo complete EMT and but rather experienced pEMT and frequently revert back into fully E phenotype (Lüönd et al., 2021). On the contrary, rarely M cells that have undergone complete EMT revert back their phenotype, rather they retain M state. Interestingly, full EMT cells are mostly found in microenvironment known to sustain dormant cancer stem cells in a static state and are thought to play a role in therapeutic resistance (Biddle et al., 2016; Lüönd et al., 2021; Brown et al., 2022). On the other side, pETM perfectly combines the high cell plasticity and stemness features required for an efficient metastatic process (Lüönd et al., 2021). Interestingly, Biddle *et al.* have developed a method to enrich a sub-population of CSCs (CD44^high^Epcam^low/−^CD24^+^) that exhibit both phenotypic plasticity and therapeutic resistance through co-treatment with TGFβ and RA (Biddle et al., 2016). Of note, such CSCs possess rapid protein turnover that could contribute to sustain the rapid cellular re-modelling occurring during transitions between phenotypic states. Therefore, the observed therapeutic resistance might be a defensive response consequent to the considerable stress caused by the rapid protein turnover itself (Biddle et al., 2016).

The connection between pEMT and stemness was first proposed by Brabletz and others who exposed the concept that migratory cancer cells derived from “stationary” epithelial cancer cells through the acquisition of a “transient EMT” in addition to stemness (T. Brabletz et al., 2005). Afterwards, in order to explain this connection, mathematical models identified the double inhibitory loops of miR-200/ZEB and LIN28/let-7 as the coupled decision-making regulatory network of EMT and stemness, respectively (Pasani et al., 2021) (Figure 2A). To this regard, LIN28/let-7 regulatory pathway is involved in the regulation of stemness genes expression by controlling key cell processes such as embryonic stem cells (ESCs) differentiation, cell growth and metabolism, somatic cell reprograming and tumor progression (Shi et al., 2008; Shyh-Chang and Daley 2013; Hikasa et al., 2016). Indeed, this pathway represents an excellent example of relationship between miRNAs (let-7) and mRNAs (LIN28). In particular, let-7 is a miRNA involved in the negative regulation of Oct4, Sox2 and Nanog gene expression therefore reducing ESC proliferation and promoting their differentiation (Li et al., 2019). On the other hand, the post-transcriptional regulation of let-7 miRNA is controlled by the conserved RNA-binding protein LIN28, which binds to 3′-UTR of let-7 and repress its maturation. As a result, LIN28 positively regulates the re-expression of stemness genes in cancer cells during the so-called “stemness windows” and, thus increasing cell proliferation by modulating a number of key molecules involved in these processes (Ma et al., 2013; F. Peng et al., 2018; Li et al., 2019).

The activation of the EMT program induces the downregulation of let-7 miRNA and the consequent upregulation of LIN28-mediated stemness gene re-expression. Intriguingly, LIN28 is also responsible of Oct4 expression tuning thus controlling stem cell fate. Indeed, only intermediate levels of Oct4 expression provides stemness whereas either very high or very low gene levels lead to loss of stem cell properties (Pasani et al., 2021). Such intermediate levels of Oct4 are generally attained by medium levels of LIN28 and let-7. Consequently, rather than E (no EMT: miR-200^high^, ZEB^low^/LIN28^low^, let-7^high^) or M (complete EMT: miR-200^low^, ZEB^high^/LIN28^high^, let-7^low^) exclusively hybrid E/M phenotypes achieve such ‘stemness window’ condition displaying medium levels of Oct4 (or LIN28) (Jolly et al., 2015b). Noteworthy, miR-200/ZEB and LIN28/let-7 can also influence each other, given that let-7 is able to target and prevent ZEB translation whereas miR200 can inhibit LIN28 translation (Pasani et al., 2021) (Figure 2A).

Therefore, EMT/MET seems to be drawing a scenario in which the balance in gene expression—rather than a direct up or downregulation—may play a crucial role in the developing of hybrid E/M state during EMP.

Recently, scRNA-seq analyses have provided encouraging confirmations to the data obtained from computational modelling. For instance, lineage tracing applying to a mouse model of metastatic pancreatic cancer revealed that the largest amount of metastatic sub clones expressed late hybrid E/M features, thus suggesting an association of this state with malignant progression (Simeonov et al., 2021). Subsequently, another study correlated the mechanism responsible for skull base chordoma cancer radioresistance with the packaging of telomere ends occurring in tumor-derived hybrid E/M cells (Q. Zhang et al., 2022). However, the majority of carcinomas are seldom composed of cells with similar phenotypical and epigenetics traits. More frequently, the heterogeneity of cancer cells itself represents an evolutive advantage able to generate cell states with various degrees of metastatic potential and resistance to various therapies (Puram et al., 2017; Baumeister et al., 2021). In particular, such heterogeneity along the EMT spectrum is emerging as a hallmark of either primary tumor and CTCs (Bocci et al., 2021).

More recently, the enhancement of stemness has been related to tissue regeneration becoming a mechanism of profound relevance in the context of non-cancer epithelial stem cell whom undergo pEMT (Lambert and Weinberg 2021). To this regard, amniotic epithelial cells (AECs), which form the innermost layer of amniotic membrane (AM), are a subset of placental stem cells which have shown to undergo EMT during pregnancy or regenerative processes (Canciello et al., 2017; Barboni et al., 2018; Canciello et al., 2020). Indeed, during pregnancy AM undergoes growth, repair, and remodeling processes due to the induction of several cycles of EMT/MET (Richardson et al., 2020). In particular, AECs transition toward M phenotype is mediated by TGFβ which triggers EMT; conversely, progesterone (P4), which is dominant pregnancy maintenance hormone, reduces EMT and partially induces MET (Canciello et al., 2017; Richardson et al., 2020). At the end of pregnancy, in preparation for labor, the rising of TGFβ (and oxidative stress) and the drop of P4 concentration lead to a significant induction of EMT and to the weakening of AM (Canciello et al., 2017; Richardson et al., 2020). Intriguingly, P4 is able to maintain E phenotype of AECs by downregulating EMT-TFs while simultaneously induces an increase in stemness, suggestive of a hybrid E/M state (Canciello et al., 2017; Mauro et al., 2019). Moreover, the induction of EMT in AECs also coincides with an increase of Nrf2 expression, thus suggesting a possible stabilization of hybrid E/M phenotype (Lollo et al., 2020). Notably, hAECs undergo *in vivo* trans-differentiation when transplanted in an injured tendon in order to taking part in mesenchymal tissue repair, therefore indicating a putative involvement/exploitation of EMP in the enhancement of AECs regenerative potential (Barboni et al., 2018).

Even adult E stem cells conserve such EMP-mediated differentiation which they adopt in order to support tissue homeostasis and regeneration. A sort of hybrid E/M polarization has been documented in stem cells of stratified epithelia such as mammary gland, prostate gland, interfollicular *epidermis* and upper airways of the lung display (Mani et al., 2008; Lambert and Weinberg 2021). In particular, these stem cells express—among the others—the master regulator of stem cells in multiple stratified epithelial tissues, ΔNp63, and several EMT-TFs including and ZEB and SLUG, even in the absence of cell-physiological stress (Newkirk et al., 2008; Chakrabarti et al., 2014; Melino et al., 2015). Intriguingly, the regenerative potential of stem cells in stratified epithelia mainly depend on the lack of a typical apical–basal polarity which permit them to rapidly trigger a pEMT program, thus acquiring hybrid E/M phenotype (Jung et al., 2019). In this scenario, EMT-related genes could play an active role in regulating the regenerative potential of these E stem cells (Lambert and Weinberg 2021). For instance, mammary stem cells (MaSCs) at the outer layer of mammary gland are the main stem cells involved in its reconstitution (Mani et al., 2008). Besides E-cadherin and ΔNp63, these E cells have been found to express, also M markers such as N-cadherin, Vimentin, Slug, and Sox9 a stemness-related TF (Lambert and Weinberg 2021). Intriguingly, using mouse-derived SLUG–yellow fluorescent protein (YFP) expressing MaSCs, was found that only the SLUG + subpopulation has the capacity for gland reconstitution following implantation in mammary fat pad, thus suggesting that the partial EMT state within whom these cells reside is fundamental for their regenerative potential (Mani et al., 2008). Moreover, transient coexpression of Slug and Sox9 induces the trnasition of differentiated luminal epithelial cells of mammary gland into MaSCs with the ability to reconstitute the mammary ductal trees (W. Guo et al., 2012). Intriguingly, both Slug and Sox9 form an autoregulatory network involved in inducing and sustaining the stemness state of MaSCs, thus suggesting that adult stem cells can adopt autoregulatory mechanisms similar to embryonic stem cells to maintain their stemness (Boyer et al., 2005; Kim et al., 2008; W. Guo et al., 2012).

Nevertheless, SLUG was also indicated as an important regulatory factor for non-epithelial stem cells such as hematopoietic, mesenchymal and muscle stem cells, suggesting that E/M plasticity and stemness may be a very ancient evolutionary association in diverse cell models (Sun et al., 2010; Torreggiani et al., 2012; P. Zhu et al., 2019; Lambert and Weinberg 2021).

Putting together this information, stemness could be considered as a function of EMP which is not necessarily always increasing during the process. Instead, stemness is likely to first increase as E cells begin to go through EMT and then decrease as they become fully M.

### Phenotypic stability factors (PFS)

Mathematical modeling studies provided insights on the multi stable nature of EMT and, in particularly, of the existence of stable hybrid E/M states. Huang et al. and Font-Clos et al. were the first showing the robust stable states of hybrid E/M phenotypes based on the topological data of EMT regulatory networks (B. Huang et al., 2017; Font-Clos et al., 2018). Subsequently, both mathematical and experimental approaches have further identified a series of molecules implied in determining and maintaining these metastable hybrid E/M intermediates. Such a proteins, called phenotypic stability factors (PSFs), can promote and stabilize hybrid E/M states and include—among the others—OVOL, GRHL2, Nrf2, ΔNP63α, NUMB, Jagged and miR-145/Oct4 (Jia et al., 2019).

The transcription factor OVOL is a well-studied regulator of embryogenesis involved in the differentiation of epidermal progenitor cells (Nair et al., 2006). During mammary morphogenesis, OVOL is expressed in terminal end bud (TEB) cells that migrate collectively forming finger-like projections. Its expression maintains TEB cells in a hybrid E/M phenotype by preventing cells that have gained partial plasticity from undergoing complete EMT (Watanabe et al., 2014). Besides, the transcription factor GRHL2 is expressed in E cells and regulates epidermal development (Shen et al., 2020). GRHL2 play a crucial role in determining E phenotype and in suppressing EMT by specifically inhibiting both ZEB1 and ZEB2 (Hari et al., 2020; He et al., 2020; Shen et al., 2020). Both OVOL and GRHL2 can operate in coupling with miR-200/ZEB thus significantly expands the range of parameters or physiological conditions controlling hybrid E/M states and/or E phenotype (Figure 2B). In this context, it has been demonstrated that OVOL upregulation can induce MET by forming a double negative feedback loop with ZEB and inhibits autocrine TGFβ (Gómez Tejeda et al., 2019; Hari et al., 2020; Pasani et al., 2021). Both GRHL2 and OVOL are considered as MET-inducing TFs since their overexpression is able to upregulate the E-cadherin levels and/or revert EMT as well as other EMT-associated traits, such as anoikis resistance, metabolic reprogramming, immune evasion, and collective cell migration (Pasani et al., 2021). On the contrary, the inhibition of OVOL or GRHL2 are able to enhance some hybrid EMT functional attributes such as tumorigenesis and stemness (Sinha et al., 2020).

Furthermore, miR-145 plays a pivotal role in inducing pEMT and stabilize hybrid E/M phenotype and, similar to OVOL and GRHL2, can drive MET when overexpressed (Chivukula and Mendell 2009; Subbalakshmi et al., 2020). Generally, miR-145 switches off pluripotency targeting the stemness factors Oct4, Sox2, and Klf4, by inducing embryonic stem cells differentiation (Chivukula and Mendell 2009; Xu et al., 2009). More in detail, miR-145 form a double negative loop with Oct4 and ZEB where the three of them mutually inhibit each other (Jolly et al., 2016) (Figure 2A). Conversely, Oct4 and miR-200 form a double positive loop where both of them induce each other (Wang et al., 2013) (Figure 2A). Therefore, miR-145/Oct4 by interacting with miR-200/ZEB form a three-way switch that enables the generation of the three diverse phenotypes (E, M and hybrid E/M).

Nrf2 has been deciphered as another important PSF for hybrid E/M forms whose constitutive expression upregulated both E-cadherin and ZEB1 in non-small cell lung carcinoma (NSCLC) and bladder cancer cells (Sinha et al., 2020). Intriguingly, mathematical modelling predicts that cells in hybrid E/M forms have higher levels of Nrf2 compared with those E or M states. This behavior is due to the fact that miR-200 and ZEB inhibit Keap1 and E-cadherin respectively, which, in turn, inhibit Nrf2 (Figure 2B). Therefore, NRF2 receives an indirect activation from both miR-200 and ZEB, thereby leading to an increase of its levels in hybrid E/M phenotype (Bocci et al., 2019b).

∆Np63α is the predominant and physiologically significant isoform of p63 in epithelial tissues (Alshammari et al., 2021). Recently, ∆Np63α has been proposed as another possible PSF since it can induce a pEMT by activating Slug (SNAIL2) as well as inhibiting ZEB *via* miR-205 (Dang et al., 2016). In particular, ΔNp63α confers a migratory phenotype through inducing a hybrid E/M state wherein components of EMT programs promote migration whereas the miR-205 maintains key E features (Dang et al., 2016). Intriguingly, P-cadherin a well-known marker of hybrid E/M phenotype is also a downstream target of ∆Np63α which, in turn, is induced by GRHL2 (Jolly et al., 2016). However, further studies are required to decipher the specific role of ∆Np63α in inducing and maintaining hybrid E/M phenotype and, more specifically, the connections existing among this transcription factor and the other members of the EMP core regulatory network.

The co-existence of E, M and hybrid E/M subpopulations in a single cell line indicates the multi-step wise organization of EMT/MET with the presence of a population heterogeneity of EMT (Jia et al., 2019). This heterogeneity is modulated at different levels and can either be generated or maintained by multiple mechanisms. Noise in the expression of involved RNAs and proteins and/or their stochastic partition at the time of cell division represent cell-autonomous mechanisms thereby cells may spontaneously undergo a phenotypic transition. Besides, the regulation of the EMP core regulatory circuit behavior due to external factors can vary from cell to cell and eventually leading to different responses in term of cell transition from one stable state to another (Jia et al., 2019). On the other hands, the population heterogeneity of EMT can be the results of cell-cell communication, as it happens for Notch-Delta-Jagged signaling.

Among the other pathways, Notch signaling is as a key regulator and mediator of cell–cell and cell-stroma communication (Jolly et al., 2015a) (Figure 2C). Notch signaling gets activated when Notch receptor on 1 cell—called Sender (S) because it sends the signal—interacts with the transmembrane ligand Delta (repressor) or Jagged (activator) on a neighboring cell, which is called Receiver (R) since it receives the signal (Boareto et al., 2015). This interaction cleaves Notch and causes the release of Notch intracellular domain (NICD) into the cytoplasm (Figure 2C). NICD can thus translocates into the nucleus and regulates the expression of Notch target genes. In particular, it activates Jagged and Notch and represses Delta (Boareto et al., 2016) (Figure 2C).

In this scenario, when Notch-Delta (N-D) interaction occurs between two neighboring cells, Notch signaling is only activated in 1 cell that becomes a Receiver (R) (high levels of Notch and low levels of Delta); conversely, Notch signaling is inhibited in the neighbor cell that hence becomes a Sender (low levels of Notch and high levels of Delta) (Boareto et al., 2015) (Figure 2D). Consequently, N-D signaling gives rise to a double negative feedback loop leading neighboring cells to adopt alternate fates (S or R) (lateral inhibition) (Boareto et al., 2016; Jolly et al., 2019) (Figure 2E).

On the other hand, when Notch-Jagged (N-J) interaction occurs, Notch signaling is activated in both neighboring cells that can both send and receive signals, thus becoming hybrid S/R cells (high Jagged and Notch levels) (Figure 2D). Consequently, N-J signaling forms a double positive feedback loop between the 2 cells that drives them to adopt a similar fate (hybrid S/R), thereby propagating the same fate across the tissue (lateral induction) (Boareto et al., 2015) (Figure 2E).

Notch signaling is deeply coupled to the EMP regulatory networks discussed in the previous sections (Figure 2C). In fact, miR-34 and miR-200 (targeting SNAIL and ZEB, respectively) reduce the levels of Notch receptor and ligands. Conversely, NICD is able to promote the transcription of SNAIL and thus acts as an EMT inducer (Jia et al., 2019). Therefore, EMT can be propagated among neighboring cells through the Notch signaling lateral induction (Jia et al., 2019). Intriguingly, the coupled of EMP and Notch core regulatory networks dramatically affect EMT outcomes despite both Delta or Jagged are able to induce EMT. In fact, N-D signaling promotes a spatial arrangement where cells in a partial or complete EMT are surrounded by E cells, whereas N-J signaling foster the formation of clusters of hybrid E/M cells (Boareto et al., 2016; Jolly et al., 2019) (Figure 2E). To this regard, Jagged1 was described as one of the most upregulated genes in collectively migrating cancer cells, suggesting that can act as an intercellular PSF that stabilizes hybrid E/M phenotype (Bocci et al., 2017; Jia et al., 2019).

Although Notch signaling pathway significantly affects EMT and its outcomes, a crucial role is played by its inhibitors. To this regard, Numb and its homologue Numb-like (Numbl) can inhibit Notch signaling whereas activated Notch signaling (NICD) inhibits Numb/Numbl, thus generating a mutually inhibitory feedback loop (Bocci et al., 2017) (Figure 2C). Recent evidences demonstrated that both Numb/Numbl can prevent the cells from undergoing a complete EMT by inhibiting Notch signaling (Bocci et al., 2017). As a result, Numb/Numbl can act as PSF for hybrid E/M phenotype increasing the percentage of hybrid E/M cells in clusters that undergo EMT (Bocci et al., 2017).

A further level of asymmetry between signaling through the ligands Delta and Jagged arises from posttranslational modifications of Notch. The glycosyltransferase Fringe decrease the affinity of Notch receptor to Jagged, thus increase its affinity to Delta ligand. Collectively, both Numb/Numbl and Fringe tend to mitigate the stimulatory N–J signaling, thus affecting the tissue patterning in a layer of cells (Boareto et al., 2015, 2016).

The nuclear factor of activated T-cell (NFATc) has been proposed as a putative non-canonical PSF for hybrid E/M phenotypes (Subbalakshmi et al., 2020). In fact, NFATc is a transcription factor which responds to Ca^2+^ signaling and regulates cell cycle progression, gene expression and apoptosis (Mognol et al., 2016). Intriguingly, it has been reported that NFATc inhibits the complete EMT and is able to preserve E-cadherin expression even in the presence of TGFβ stimulation (S. K. Singh et al., 2015; Gould et al., 2016). Moreover, NFATc can also activate Sox2 transcription, thus leading to the upregulation of ALDH expression (a well-known stemness marker) (S. K. Singh et al., 2015; Xiao et al., 2017). However, unlike the other canonical PSFs, stochastic simulations have demonstrated that NAFTc is not able to maintain cells in hybrid E/M state, but instead it enables the co-existence of E, M and hybrid E/M phenotypes (Subbalakshmi et al., 2020).

All together these findings demonstrated how PSF modulation of hybrid E/M states is integrated with the EMT decision-making circuit represented by miR-200/ZEB. As consequence, the connections among these genes are indeed larger than expected and there is also an increased complexity of the physiological conditions under which a hybrid E/M state can be attained. Intriguingly, since miR-200/ZEB network is strictly related with stemness circuit LIN28/let-7, also PSF modulation can affect plasticity and stem properties (Jolly et al., 2015b). Therefore, PSF coupling can create a region of parameter space in which the only stable state is a hybrid one.

### Collective migration and migratory plasticity

Like stemness, EMT is regulated by fundamental embryonic pathways including Wnt, TGFβ, HIF1α, HGF and NF-κB which are also involved in the development of migratory abilities and behavior (T. Brabletz et al., 2005). During this process, cells can undergo a partial or complete EMT and thus move collectively or individually through the ECM or in the bloodstream (B. Huang et al., 2015).

Collective migration is reflective of the hybrid E/M phenotype (Jia et al., 2019). Collectively migrating cells display both E (cell-cell adhesion) an M (migration) properties that allow them to invade the bloodstream as clusters rather than single cells (individual migration). To this regard, CTCs collectively moving as clusters bear higher metastatic potential respect to individual CTCs formed by fully M cells that have completed EMT process (Jia et al., 2019).

Collective migration is a key process during the development of most organisms and involves either M cells making dynamic contacts and frequently changing neighbor cells or E cells that typically display more stable cell-cell interactions (Weijer 2009). As consequence of gradients of extracellular signaling molecules, E cells undergo partial or complete EMT to reach distant sites during gastrulation or outmigration from the neural crest (Lambert and Weinberg 2021). Intriguingly, it has been demonstrated that during the development, the persistence of E-cadherin expression is required to prevent the disassembly of the migrating stem cell clusters (Plygawko et al., 2020). However, recent findings indicate that usually only leader stem cells with hybrid E/M traits sense the external signal gradient and initiate the migration, and that adjacent cells follow through strong cell-cell contacts (Weijer 2009).

During re-epithelialization, epidermal cells undergo pEMT showing hybrid E/M features and collective migration as they head towards the wound site. This process also involves a transition to a stem-like state, thus indicating that it may be a common feature of regenerative responses (Lambert and Weinberg 2021). During wound-healing response, cells at the leading edge of these advancing mass head to the wounded area. These cells bear the most prominent M features and are non-proliferative (Lambert and Weinberg 2021). On the other hand, the leading edge is immediately followed by the front of actively proliferating cells that reside in the so called-proliferative hub. These cells are more E-like and are instead involved in the restoration of proper cell numbers (Lambert and Weinberg 2021).

In addition to chemical communication, the stiffness of ECM also plays a key role in regulating EMT. In fact, any alternation in ECM stiffness induce multiple signaling pathways including ZEB-LOXL2, HA-CD44, PI3K/Akt and YAP/TAZ that eventually can trigger EMT (Dupont et al., 2011; Leight et al., 2012; D. H. Peng et al., 2017; Razinia et al., 2017; Ondeck et al., 2019; Matte et al., 2019). However, cells undergoing EMT can in turn regulate ECM in order to enable and promote collective cell migration (Jia et al., 2019). Indeed, cancer cells—behind the leading of cancer-associated fibroblasts (CAFs)—induce matrix remodeling in order to create track into ECM within which migration can properly occur (Sinha et al., 2020). To this aim, anterior CAFs creates micro-tracks by secreting membrane type 1 metalloprotease (MMP1) that promote the initial single-cell migration. Afterwards, lateral ECM interfaces undergo a large-scale degradation by means of other proteases that enable the following collective migration. In this process, adherent junctions play a fundamental role to allow collective migration of cluster of cells (Jia et al., 2019). Recently, it has been reported that both CAFs and cancer cells undergo collective migration through the heterophilic adhesion of E-cadherin and N-cadherin with similar affinity as that homophilic E-cadherin interactions (Kai et al., 2018; Tiwari et al., 2018). Therefore, cancer cells with hybrid E/M features which are sited at the junction between tumor hive and ECM, express E-cadherin on their membrane through which they interact with N-cadherin on the CAF membrane in order to propagate and promote fibroblast-led collective cancer cell migration (Sinha et al., 2020).

**FIGURE 3:**
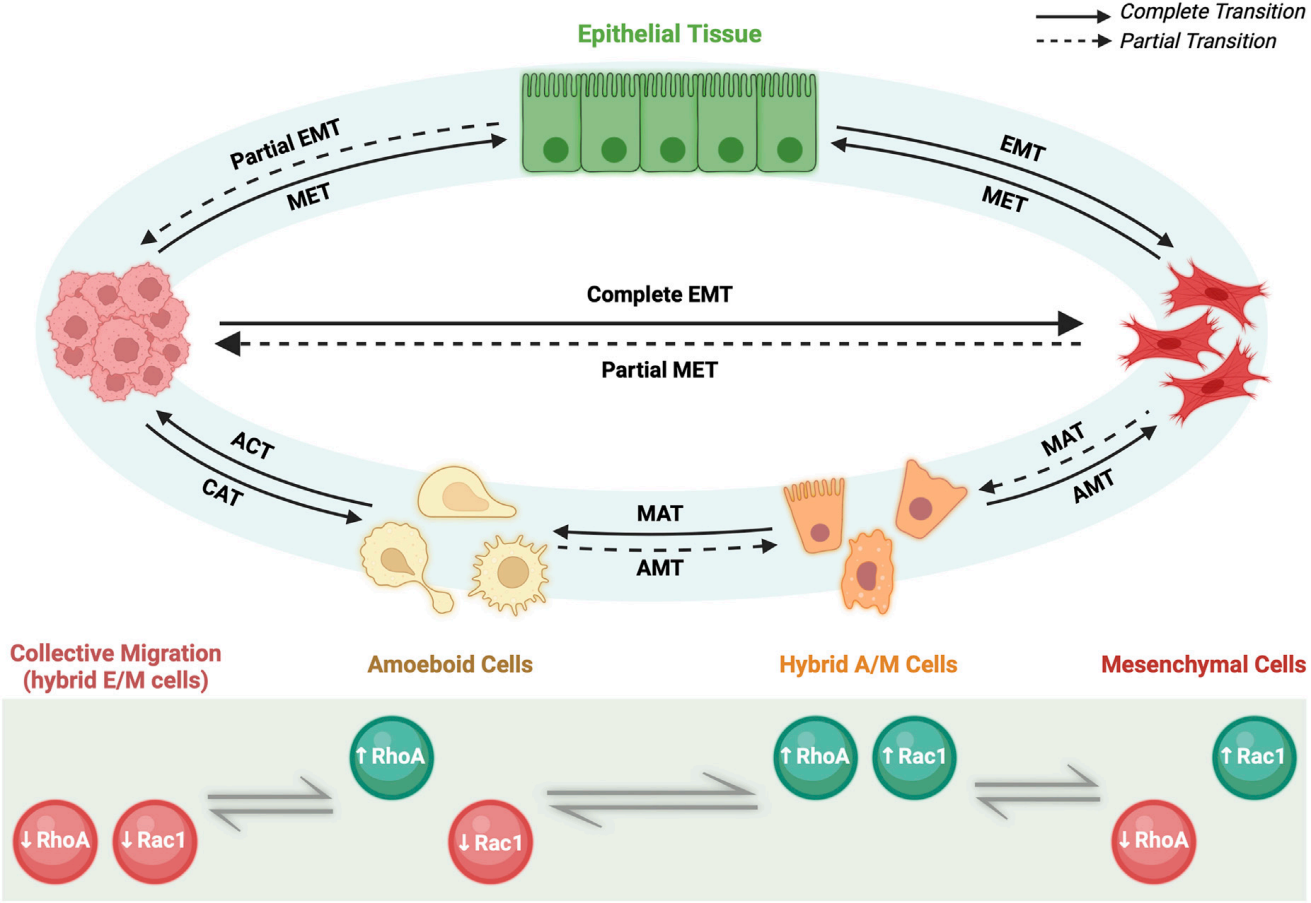
Migratory plasticity. Primary epithelial tissue through partial EMT (pEMT) and complete EMT can give rise to motile cells capable of invasion *via* collective migration of hybrid E/M cells or individual migration of mesenchymal cells, respectively. Afterwards, specific microenvironment conditions govern reversible cell fates. On one hand, cluster of hybrid E/M cells can undergo collective-individual transition either by completing EMT process and becoming mesenchymal cells or *via* collective-amoeboid transition (CAT) and giving rise to amoeboid cells. The reverse process is called amoeboid-collective transition (ACT). Amoeboid and mesenchymal cells can reversibly interchange phenotype *via* amoeboid-mesenchymal (AMT) and mesenchymal-amoeboid transition (MAT), respectively. Partial AMT and partial MAT give rise to hybrid A/M cells with mixed amoeboid and mesenchymal features. On the other hand, mesenchymal cells *via* pEMT can either undergo individual-collective migration giving rise to a cluster of migratory hybrid E/M cells or revert their phenotype *via* MET and becoming epithelial cells. All the transitions rely on the levels of two small GTPases, RhoA and Rac1, which can be upregulated (green) or downregulated (red) depending on the cell phenotype.

Notably, depending on the nature of the interactions between the leading and the following cells it is possible to distinguish between two types of collective migration: 1) collective migration of tight fasten epithelial cells bearing stable cell-cell homophilic interactions among, desmosome, adherent, tight, and gap junctions 2) collective migration of mesenchymal cells bearing weak heterophilic cadherin interactions to maintain the leader–follower coordination (Mercedes et al., 2021). However, collective migration is representative of whole array of the individual migrations simultaneously acting within a packed cluster of cells (Figure 3). Basically, individually migrating cells can show at least two distinct migrating phenotypes: amoeboid (A) or mesenchymal (M). Cells in M phenotype, as such as those whom undergone a complete EMT, have lamellipodia and/or filopodia on their leading edge and secrete MMPs to remodel and degrade the ECM, thus acting as “path generator”. On the contrary, cells in A phenotype—that do not secrete MMPs—are round-shaped with blebby structures on their leading edge and tend to squeeze into ECM gaps, acting rather as “path finders” (B. Huang et al., 2015). Spontaneously or under the influence of external microenvironment, cancer cells are able to toggle between A and M migrating phenotype by undergoing Amoeboid to Mesenchymal Transition (AMT) or Mesenchymal to Amoeboid Transition (MAT) (B. Huang et al., 2015) (Figure 3). However, it is now evident that cancer cells could bear mixed A and M characteristics, indicative of a hybrid A/M phenotype (Sahai and Marshall 2003; B. Huang et al., 2015). Such a migratory hybrid A/M phenotype is characterized by cells adopting either lamellipodia and blebs protrusions on their leading edge. Those hybrid A/M cells have high grade of plasticity that allows them to adapt rapidly to the microenvironmental changes (B. Huang et al., 2015).

Recently, it has been demonstrated that the transition among the three individual migratory phenotypes (A, M and hybrid A/M) is regulated by the so-called AMT/MAT core regulatory circuit (Figure 2A). Such regulatory network is formed by the double inhibitory loop acting between RhoA and Rac1, two small GTPases involved in a number of signaling pathways including membrane and actin remodeling (B. Huang et al., 2015; Salloum et al., 2020). In detail, RhoA/Rac1 circuit determines a three-way switch among these three stable migratory states: A (high RhoA-GTP, low Rac1-GTP), M (low RhoA-GTP, high Rac1-GTP), and hybrid A/M (high RhoA-GTP, high Rac1-GTP). In this scenario, the A migrating phenotype is characterized by elevated actomyosin contractility due to the high levels of active RhoA; conversely, M migrating cells show elevated actin polymerization induced by the high levels of active Rac1; as consequence, hybrid A/M phenotype that displays mixed A and M features, has comparable level of both actin polymerization and actomyosin contractility due to the high levels of both active RhoA and Rac1 (B. Huang et al., 2015).

Intriguingly, it has been proposed that miR-34/SNAIL and miR-200/ZEB circuits could govern migratory plasticity by regulating RhoA and Rac1, thus revealing an association between EMT/MET and AMT/MAT core regulatory networks (B. Huang et al., 2015) (Figure 2A). In particular, both miR-34 and miR-200—gatekeepers of epithelial states—act as inhibitors of either RhoA and Rac1 GTPases, thus interfering with cells migration. The inclusion of EMT circuit into the migratory circuit can potentially give rise to novel dynamics. In fact, miR-34/miR-200 circuit induces a further level of complexity at the RhoA/Rac1 equilibrium made of three steady states (A, M and hybrid A/M): considering that both miR-34 and miR-200 inhibit the activation of either RhoA and Rac1, it has been taken in consideration the existence of a fourth possible state, where both GTPases are simultaneously downregulated. Therefore, the incorporation of the microRNAs signaling into the RhoA/Rac1 circuit led to the generation of a four-way switch among these four possible states: 1) high RhoA, low Rac1 (Ameboid); 2) low RhoA, high Rac1 (Mesenchymal); 3) high RhoA, high Rac1 (hybrid A/M migration) and, finally, 4) low RhoA, low Rac1 (Figure 3). It is worth to note that this forth steady state was determined by mathematical models. Intriguingly, the low RhoA/low Rac1 state has been proposed to be associated with hybrid E/M phenotype and consequently with the collective migration (B. Huang et al., 2015). To this regard, there are experimental evidences that demonstrated that moderate levels of both active RhoA and Rac1 promote wound healing, a process involved collective migration of hybrid E/M forms (Desai et al., 2004). Furthermore, moderate levels of RhoA or Rac1 have been reported to induce hybrid E/M phenotype in different tumor models and to promote metastatic process (Makrodouli et al., 2011; Kröger et al., 2019; Carstens et al., 2021). Consistently, collective migration during early development in *Drosophila* have been found related to moderate expression levels of both RhoA and Rac1 (Geisbrecht and Montell 2004).

The association between EMT/MET and AMT/MAT core regulatory circuits would help to explain the existing interplay between collective and individual migration (B. Huang et al., 2015). To this regard, recent findings has reported that cluster of migrating tumors cells can switch between collective (hybrid E/M) to individual (A, M or hybrid A/M) type of migration and *vice versa* (Friedl and Wolf 2010; B. Huang et al., 2015) (Figure 3). Therefore, the terms Collective to Mesenchymal Transition (CMT) and Collective to Ameboid Transition (CAM) are generally used to indicate transition towards M and A migrating phenotype, respectively. For this reason, deciphering the molecular mechanisms governing the interconversion between collective and individual type of migration would be critical to understand deeply how EMP works.

### Metabolic reprogramming and metabolic plasticity

Cellular metabolism regulates biochemical processes and is profoundly influenced by intracellular and extracellular changes. In particular, cells undergoing EMT encounter complex changes during the cellular transition. As consequence, cellular metabolism faces a fine-tuned modulation in order to meet the increased bioenergetic demands (Ramesh et al., 2020). Moreover, cells undergoing EMT typically become more resilient to intracellular and extracellular stresses and, in the case of tumor cells, they become resistant either to therapeutic treatments and to facing different microenvironment in distant metastatic niches (Jia et al., 2019).

Metabolic reprogramming is an emerging hallmark of cancer and is fundamentally related to the EMT-induced changes. In particular, the Warburg effect or aerobic glycolysis, is the most recognized metabolic phenotype observed in cancers (Hua et al., 2020). This process takes place in the cytosol and consists in the shifting of cellular metabolism form one mainly based on the oxidative phosphorylation (OXPHOS) towards another that relies on glycolysis even under aerobic conditions (Hua et al., 2020). Therefore, the Warburg effect induces the upregulation of glucose uptake in cancer cells as bioenergetic fuel, even though the ATP yield of glycolysis is very inefficient (2 mol of ATP per mol glucose compared obtained from glycolysis compared to 36 mol of ATP per mol obtained from OXPHOS) (Hua et al., 2020). However, tumor cells seem to experience several advantages from this shifting of metabolic phenotype. In fact, a cell metabolism mainly based on aerobic glycolysis better satisfies the fast bioenergetic demand of rapidly proliferating cancer cells (Kroemer and Pouyssegur 2008; Heiden et al., 2009; Cairns et al., 2011). Moreover, enhanced glycolysis accompanied by increased lactate fermentation and alleviated mitochondrial respiration significantly increases cancer cells protection against oxygen fluctuations and reactive oxygen species (ROS) (Hua et al., 2020; Jia et al., 2021).

To this regard, TGFβ increases O_2_^•-^ production by mitochondria which in turn stimulates EMT. The mitochondrial enzyme superoxide dismutase 2 (SOD2) catalyzes the conversion of O_2_^•-^ in H_2_O_2_ (Rhyu et al., 2005; Kinugasa et al., 2015). This effect is counterbalanced by mitochondrial thioredoxin (TXN2) that simultaneously represses high-mobility group AT-hook 2 (HMGA2) activity, thus inhibiting TGFβ-induced EMT (Ishikawa et al., 2014). Therefore, TGFβ induces EMT by actively stimulating ROS production and simultaneously keeps their concentration within a not harmful range for cells by inducing antioxidant enzymes. An additional source of TGFβ-induced ROS is NADPH oxidase 4 (NOX4), localized at the cell membrane, which stimulates receptor tyrosine kinase-induced p38MAPK signaling, thus enhancing EMT by promoting SNAI1 expression (Mondol et al., 2014).

The Warburg effect is also characterized by the accumulation of lactate—the final product of glycolytic process—that significantly contributes to increase tumor acidity (Hua et al., 2020). Intriguingly, cancer cells can use lactate as energy source and also metabolize those lactate molecules produced by the cells residing in the tumor microenvironment, thus promoting the formation of an acidic environment. To this regard, lactate increases during cancer metastasis and it is reported to induce apoptosis resistance, survival, proliferation, immune escape and tumor invasion through TGFβ regulation of SNAI and MMP2 (Hua et al., 2020).

Recent findings further indicate that metabolic changes and EMT are intertwined. In fact, metabolic alterations can possibly induce EMT as well as EMT may lead to metabolic changes. To this regard, TGFβ promote glycolysis and induce the upregulation of glucose transporters (GLUTs), hexokinase 2 (HK2), lactate dehydrogenase A (LDHA) and 6-phosphofructo-2-kinase/fructose-2,6-biphosphatase 3 (PFKFB3) and expression (Rodríguez-García A et al., 2017; Ramesh V et al., 2020). Similarly, it was demonstrated that TGFβ also induce fatty acid β-oxidation (FAO), thus increasing the rate of energy production (Jiang et al., 2017). Recent studies also investigated the specific role of EMT-TFs in inducing metabolic changes regardless by TGFβ stimulation. In particular, as expected, SNAIL and ZEB were found downregulate OXPHOS and upregulate glycolysis whereas SLUG and TWIST can also inhibit mitochondrial respiration (Dong et al., 2013; Krebs et al., 2017; Røsland et al., 2019). In addition, ZEB can directly promote glucose uptake by transcriptionally activating GLUT3 and the synthesis of long-chain polyunsaturated fatty acids, eventually inducing ferroptosis (Masin et al., 2014; Viswanathan et al., 2017). Finally, both miR-200 and miR-34, epithelial gatekeepers, play instead an opposite role by targeting LDHA, thereby repressing glycolysis (Kaller et al., 2011; Yuan et al., 2017).

It is worth to note that the switching toward mainly glycolytic metabolism is a physiological adaptive developmental program that can be aberrantly activated in cancer (Jia et al., 2021). In fact, several types of non-cancer cell also exhibit such a metabolic reprogramming. For instance, glycolysis is mighty used by adult stem cells that reside in hypoxic niches in order to maintain their self-renewal capacity. Similarly, during gastrulation, neural crest cells use aerobic glycolysis as they undergo EMT to initiate migration through the embryo (Jia et al., 2021).

As discussed for the EMT and migration processes, also metabolic reprogramming is far to be a binary decision-making process in which cells can alternatively use only glycolysis or OXPHOS. Indeed, it has been demonstrated that hybrid E/M cancer cells usually adopt a mixed metabolic phenotype characterized by high rates of both glycolysis and OXPHOS in order increase their metastatic potential and induce drug resistance (Jia et al., 2019, 2021). In particular, it is generally accepted that E cells metabolism mainly depend on OXPHOS (Zu and Guppy 2004; Krapf et al., 2020). Upon EMT induction, E cells experience an increase of their glycolytic rate as they pass through hybrid E/M intermediates (OXPHOS^high^/glycolysis^high^). At this stage, hybrid E/M cells can either decrease OXPHOS to complete transition into quiescent M cells (glycolysis^high^) or decrease glycolysis and lose stemness and eventually complete the transition into the differentiated M cells (OXPHOS^high^) (Colacino et al., 2018; Luo et al., 2018).

To this regard, the high rate of cancer cell metabolism increases mitochondrial mass and activity and can ultimately confer stem-like characteristic, thus promoting drug resistance and metastasis (Jia et al., 2021). Intriguingly, another important parallelism with hybrid E/M cells derived from the observation that cancer cell during collective migration exhibit metabolic coordination where leader cells use preferentially OXPHOS whereas the adjacent cells use more glycolysis (Jia et al., 2021). Supporting the hypothesis that mixed metabolic phenotype is a hallmark of hybrid E/M phenotype, it has been reported that the maximum levels of Nrf2 (PSF of hybrid E/M cells) were found in mouse breast cancer-derived CTC showing elevated rate of both OXPHOS and glycolysis (mixed metabolic phenotype) (Lebleu et al., 2014; Bocci et al., 2019a). Concurrently, hybrid E/M breast cancer stem cells—which are positive for the stemness marker aldehyde dehydrogenase (ALDH)—have been reported to show higher OXPHOS and similar glycolysis rate, thus indicating a mixed metabolic phenotype (Colacino et al., 2018; Luo et al., 2018). However, although the studies regarding a link of hybrid E/M with mixed metabolic phenotype are very promising, further experiments aimed to directly assess the metabolic character of hybrid E/M cells are still required in order to claim a direct connection. Moreover, further advances in single-cell multi-omics (e.g. transcriptomics and metabolomics) profiles will help to understand the phenotype–metabolism plasticity coupling.

### Immunosuppressive traits of hybrid E/M states and immune plasticity

The hybrid E/M state has recently been found to modulate the immune system through an intricate crosstalk between hybrid and immune cells, thus influencing biological processes such as invasion, metastasis and regeneration. In particular, hybrid E/M state in cancer cells is associated with immune evasion and suppression. Classically, cancer cells are primarily recognized by the immune system by the expression of tumor-associated antigens (TAAs). Soluble TAA is taken up by antigen presenting cells (APCs)—such as dendritic cells (DCs)—which present this antigen to a cytotoxic T lymphocyte (CTL) bearing specific T cell receptor (TCR) for this TAA, triggering CTL activation. However, all nucleated cells—including cancer cells—express MHC class I in order to present endogenous antigens to the immune system (Mullins et al., 2022). Once activated, CTLs migrate to the tumor, bind to a TAA presented on MHC class I, and lyse the cancer cell, thus initiating immune response.

**FIGURE 4:**
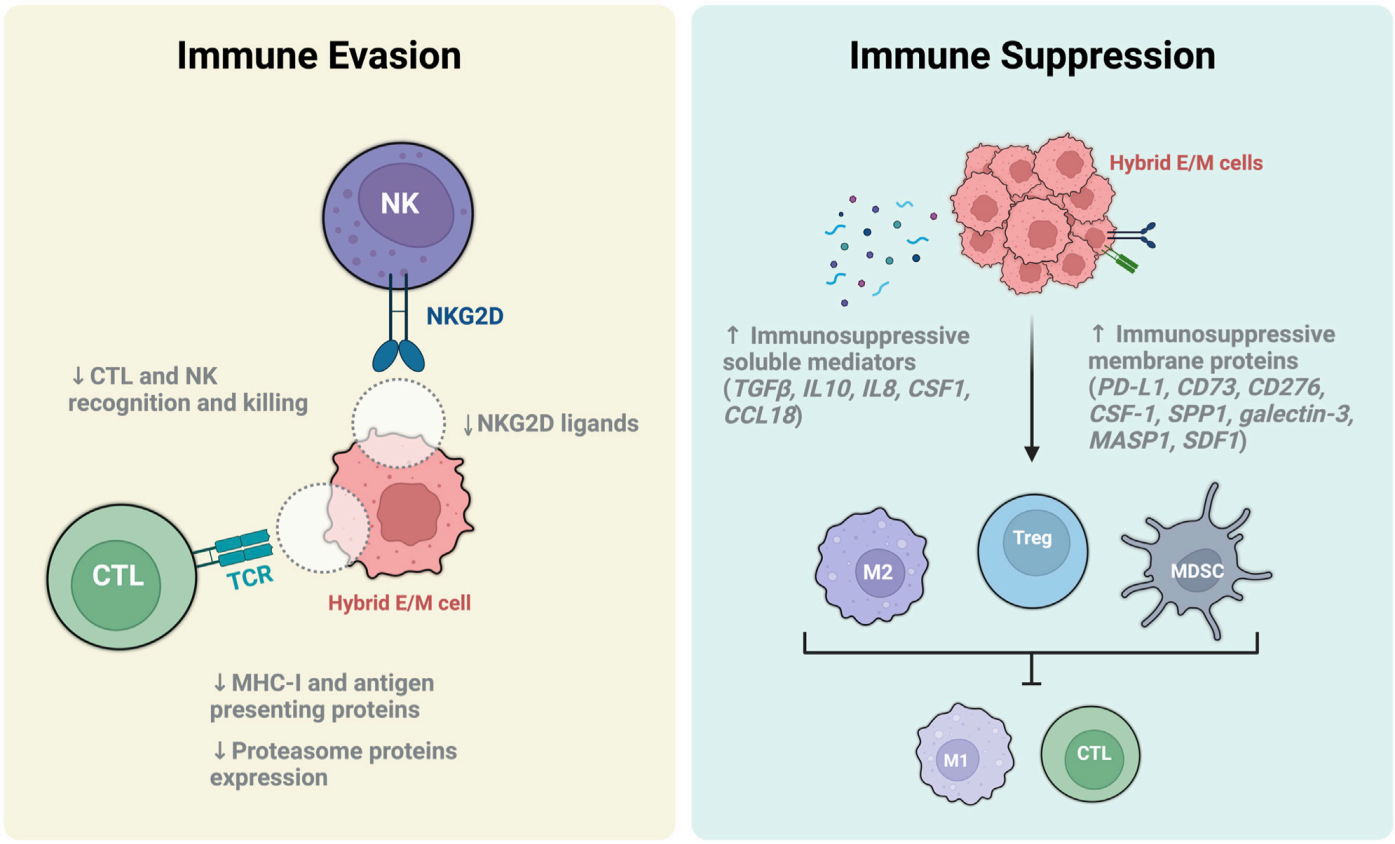
Immune escape and immune suppression mechanisms of hybrid E/M cells. Immune evasion is a mechanism thereby hybrid E/M cells can elude immune surveillance and killing by cytotoxic T lymphocyte (CTL) and Natural killer cells (NK). The main mechanisms involved include the downregulation of T cell receptor (TCR) and NKG2D ligands on hybrid E/M cell surface, the inhibition of the antigen presenting machinery and proteasome protein expression (light yellow box). On the other hand, hybrid E/M cells perform the immune suppression by increasing the release of immunosuppressive soluble mediators, upregulating the expression of immunosuppressive membrane proteins and inducing the differentiation of immune cells toward their immunosuppressive phenotypes such as anti-inflammatory M2 macrophages, regulatory T cells (Treg) and myeloid-derived suppressor cells (MDSC) (light blue box). These cells, in turn, have the ability to damp the immune response by inhibiting the activation of immune stimulatory cells (e.g., M1 macrophages, CTL), thus promoting the formation of an immunosuppressive microenvironments.

However, a persistent immune response induces phenotype instability on cancer cells which start to produces several phenotype clones able to evade immune recognition (Mullins et al., 2022). Generally, the mechanisms through which cancer cells evade immune surveillance include, among the others, the downregulation of MHC class I and other antigen presentation proteins, the upregulation of immunosuppressive membrane proteins, the production of soluble mediators able to create and immunosuppressive microenvironment and the polarization of immune cells toward a more tolerogenic phenotype such as regulatory T cells (Tregs), myeloid-derived suppressor cells (MDSCs) and anti-inflammatory macrophages (M2 macrophages) (Mullins et al., 2022) (Figure 4).

During the first stages of tumor progression, circulating monocytes are recruited into the tumor microenvironment from circulation where they are converted into tumor-associated macrophages (TAMs) (Zhou et al., 2020). In this site, activated TAM polarize into M1 inflammatory macrophages and elicit an anti-tumor activity by promoting CTL-mediated tumor elimination. However, at later stages, TAMs and Th cells polarize into M2 macrophages and Tregs respectively, and suppress the activation of anti-cancer immune cells (e.g. M1 macrophages and CTLs) also inducing tumor tissue remodeling and angiogenesis (Sica and Mantovani 2012) (Figure 4).

Intriguingly, EMP and immune evasion has been found profoundly correlated since many EMT-related actors also play a critical role in damping the immune response, thus promoting cancer growth and metastasis (Mullins et al., 2022). To this regard, several *in vivo* experiments with mouse models have demonstrated that tumor formed by EMT-derived M-like cells show a reduced number of tumor-suppressing cells and an increased infiltration of tumor-promoting cells, thus suggesting a role of EMP in mediating immunosuppression (Kudo-Saito et al., 2009). In addition, it has been recently found that SLUG may directly induce the downregulation of MHC class I and other antigen presenting proteins, thus preventing the recognition of cancer cells by CTLs (Antony and Huang 2017; Dongre et al., 2021). Nonetheless, other EMT-TFs such as brachyury and mucin 1 (MUC1) are able to induce resistance to natural killer (NK) and CTL cytotoxicity (David et al., 2016; Rajabi and Kufe 2017).

Recently, it has been demonstrated that hybrid E/M cells induce the formation of a tumor-promoting microenvironment through the upregulation of potent immunosuppressive proteins, including PD-L1, CD73 (5′-NT), CD276 (B7-H3), CSF1 (M-CSF), SPP1, galectin-3, MASP1, and SDF1 (CXCL12) (Kudo-Saito et al., 2009; Dongre et al., 2021; Mullins et al., 2022) (Figure 4). Among the others, PD-L1 was found particularly associated to hybrid E/M states and tumor evasion (Sahoo et al., 2021b). To this regard, several studies have extensively demonstrated the association of PD-L1 with tumors displaying heterogeneous hybrid E/M phenotypes and malignant progression (Asante et al., 2021). Moreover, the epithelial gatekeeper miR-200 was found to inhibit PD-L1 expression in E cells whereas ZEB directly induces its upregulation during pEMT along with CD47 (or IAP), a surface protein that promotes tumor associated macrophages (TAM) polarization to a M2-like tumor-promoting phenotype (L. Chen et al., 2014; Sahoo et al., 2021b). To this regard, hybrid E/M and M cells show a comparable high expression of PD-L1 respect to E cells, thus indicating that hybrid E/M cells adopt an M-like immunosuppressive trait without losing the plasticity advantageous (Sahoo et al., 2021b). Noteworthy, the fact that hybrid E/M cells preferentially retain well-defined selective advantages from either E or M cells could suggest—fascinatingly—a finer reprogramming than previously expected. Another mechanism through which hybrid E/M cancer cells induce immune evasion is related to the production of soluble factors. In particular, it has been reported that hybrid E/M cells upregulate the production of immunosuppressive mediators such as TGFβ, IL-8, IL-10, CSF1 (M-CSF), and CCL18 (Taki et al., 2018; Y. Guo, Xie, and Luo 2022) (Figure 4). These molecules are able to induce tolerogenic polarization of Tregs, MDSCs and M2 macrophages, and also to recruit CAFs which in turn participate to generate a tumor-promoting microenvironment (Mariathasan et al., 2018; Mullins et al., 2022). Nevertheless, tumor associated immune cells are able to produce an array of secreted factors which in turn induce and maintain the hybrid E/M phenotype, thus creating a positive feedback loop (Fan et al., 2014; Puram et al., 2017; Aggarwal et al., 2021). Intriguingly, it has been recently reported the presence of a hybrid E/M population of tumor-specific keratinocytes (TSKs) in human cutaneous squamous cell carcinoma (Ji et al., 2020). Intriguingly, the TSK cells expressed integrins ITGA3 (a well-known hybrid E/M marker) and the immunosuppressive B7-H3 molecule (Ji et al., 2020). These hybrid E/M cells are perfectly integrated in tumor microenvironment and works in concert with TAM, CAF and MDSCs to secreted immunosuppressive factors, polarize immune cells and elude immune surveillance (Mullins et al., 2022) (Figure 4).

Furthermore, the elevated plasticity of hybrid E/M states confers an evolutionary advantage related to an increased therapy resistance, thus demonstrating the role of non-genetic (perhaps phenotypic) adaptations in enhancing toleration to drug treatments (Sahoo et al., 2021a). Intriguingly, it has been proposed that such non-genetic heterogeneity could foster a collective survival of hybrid E/M cells even in the absence of any direct cell–cell cooperation, despite of the original sensitivity of the individual cells (Sahoo et al., 2021a). Similarly, another independent study highlighted the ability of cancer cells with hybrid E/M phenotypes to acquire a selective resistance to ionizing radiations (Zolghadr et al., 2021). Noteworthy, it has been reported that the treatment with ionizing radiations pushed hybrid E/M cells towards complete EMT. In particular it seemed to exist an association between the hybrid E/M-to-fully M remodeling and the increased therapy resistance, further highlighted the importance of hybrid E/M cells plasticity in the adaptation to adverse conditions (Zolghadr et al., 2021).

Tumor microenvironment is of fundamental importance for metastatic potential hybrid E/M cancer cells and actively participate to ease the migration toward the second site of colonization. In particular, the spatial localization of hybrid E/M cells at the invading edge of tumor facilitate the crosstalk among hybrid E/M cells, tumor associated cells and normal epithelium (Mullins et al., 2022). To this regard, CAFs play an important role in the induction and maintenance of plasticity in hybrid E/M cells by secreting TGFβ, latent TGFβ binding proteins (LTBPs), SDF1 (or CXCL12) and FGF (Dongre et al., 2017; Aggarwal et al., 2021; Bagati et al., 2021). On the other hand, hybrid E/M cells secrete TGFβ and TNFα that acts on normal epithelium to induce EMT, thus subverting the normal histological architecture and facilitating the lateral invasion of small cluster of hybrid E/M cells (P. Singh et al., 2021). To this regard, when hybrid E/M cells migrate in clusters they decrease the expression of NK activating ligands (such as NKG2D), thus preventing their recognition and lysis (Labelle et al., 2011; Lo et al., 2020; Lambert and Weinberg 2021) (Figure 4).

The decrease of MHC class I presentation is a hallmark of EMP and is associated with the induction of hybrid E/M phenotype and immune evasion (Figure 4). Generally, peptides loaded and presented on MHC class I primary derived from the degradation of cytosolic proteins (e.g. self-antigens, viral peptides and TAA) in the proteasome through a process regulated by IFNγ. Importantly, hybrid E/M states heve been also associated with a downregulation of proteasome protein expression (Tripathi et al., 2017). In particular, the aberrant MHC class I presentation in hybrid E/M cells was found related to a dysregulation of STAT signaling caused by an increase of phospho-STAT3 and a decrease of phospho-STAT1. In fact, STAT1 induces NLR family CARD domain containing 5 (NLRC5) protein which is the master transcription factor regulating the expression of MHC class I genes and proteasome subunits (Kobayashi and Van Den Elsen 2012; Yoshihama et al., 2016).

Notably, hybrid phenotypes manifestation is not limited to EMP-related process (Jia et al., 2019). Naive CD4^+^T cells are thought to have alternative cell fate decisions between Th1 and Th2 lineages (J. Zhu and Paul 2010). To this regard, IFNγ and IL12 *via* STAT1 and STAT4 signaling respectively induce Th1 differentiation by activating T-bet transcription factor. Conversely, Th2 differentiation is induced by IL-4 *via* STAT6 signaling by activating the GATA-3 transcription factor (Swain et al., 1990; Zheng and Flavell 1997; Szabo et al., 2000; Afkarian et al., 2002). However, recent studies revealed that differentiated CD4^+^T cells can adopt a mixed Th1/2 phenotype (S. Huang 2013; Peine et al., 2013). These bifunctional T-bet^+^GATA-3^+^ hybrid Th1/2 cells integrate the Th1-derived IFNγ and IL12 signals together with Th2-derived IL-4 signals. Therefore, hybrid Th1/2 cells can either support an inflammatory type-1 and type-2 immune responses but causing significantly less immunopathology compared to Th1 and Th2 cells alone (Peine et al., 2013). Intriguingly, hybrid phenotype is stably maintained in memory cells and resists to differentiation into Th1 or Th2 cells induced by the relative promoting stimuli, which rather generate a modulation of the combined Th1/2 response without abolishing either (Peine et al., 2013).

### Open questions and future perspectives

In conclusion, here is summarized the literature demonstrating the existing link among EMP program with stemness, migration, metabolism and immune properties of normal and neoplastic epithelial cells. Although a number of increasing works reported a fine regulated and intertwined plan for cell plasticity, there is still lack of a clear picture of biological mechanisms underlying this link. However, the involvement of hybrid E/M phenotypes in a tremendous number of different biological processes makes now clear how important these hybrid states are for either physiology and pathology. On that account, uncovering the mechanisms regulating hybrid cell plasticity with unbiased biological models will be fundamental to determine how these plasticity programs are adopted by normal cells or aberrantly corrupted during tumorigenesis. Nonetheless, it is somehow evident that the majority of the studies regarding hybrid E/M states and their biology have been performed in cancer models. Unfortunately, even though these models represent a valuable source of hybrid E/M cells and, particularly, the sole possibility to study and test alternative treatments and therapies to combat cancer progression, they do not depict the global picture. In fact, the EMP mechanisms and the generation of hybrid E/M phenotypes in cancer cells represent a deregulation of what physiologically happens in non-cancer cells during process such as embryo development, differentiation and tissue repair. For this reason, studying hybrid E/M states in non-cancer models (e.g. fetal and adult stem cells) would remarkably help the understanding of their physiological behavior, thus improving the comprehension of their pathological repercussions. Noteworthy, the generation of hybrid E/M states is accompanied by massive changes not only in the phenotype but also in the whole cell biology including transcription, protein expression, cell-cell communication, metabolism, migration and immunomodulatory properties. Such complexity is often challenging to evaluate with discrete approaches. Therefore, computational modeling is unceasingly becoming an invaluable tool for the study of this process due to the possibility to create artificial networks based on biological dataset and to predict the possible scenarios, thus helping to understand phenomena. Moreover, computational modeling can manage complexity across multiple levels of analysis, allowing data to be integrated and related each other in a way that is not always straightforward. Anyway, such predictive models could often be too simple or reductionistic respect to its biological counterpart, thus not capturing all of the relevant details and leading to misinterpretations. Therefore, it is crucial selecting the proper omics approaches as inputs for the computational analyses in order to try to resolve some of these issues. To this regard, scRNA-seq represents a powerful resource providing individually insights into the existence and behavior of different cell types, particularly useful in the field of hybrid E/M states. On the other side, most of the cell decisions are taken at protein level; thus, building notions relying solely on transcriptomic information could represent a limitation. Therefore, the integration of proteomics (and sometimes lipidomics) approach could help to depict a more accurate picture.
